# Quantitative PCR for leprosy diagnosis and monitoring in household contacts: A follow-up study, 2011–2018

**DOI:** 10.1038/s41598-019-52640-5

**Published:** 2019-11-13

**Authors:** Fernanda S. N. Manta, Raquel R. Barbieri, Suelen J. M. Moreira, Paulo T. S. Santos, José A. C. Nery, Nádia C. Duppre, Anna M. Sales, Antônio G. Pacheco, Mariana A. Hacker, Alice M. Machado, Euzenir N. Sarno, Milton O. Moraes

**Affiliations:** 1 Laboratório de Hanseníase, Instituto Oswaldo Cruz - Fiocruz, Rio de Janeiro, RJ Brasil; 2 Programa de Computação Científica (PROCC), Fiocruz, Rio de Janeiro, RJ Brasil

**Keywords:** Molecular biology, Bacterial infection

## Abstract

Household contacts (HHC) of leprosy patients exhibit high-risk of developing leprosy and contact tracing is helpful for early diagnosis. From 2011 to 2018,2,437 HHC were examined in a clinic in Rio de Janeiro, Brazil and 16S qPCR was used for diagnosis and monitoring of contacts. Fifty-four HHCs were clinically diagnosed with leprosy at intake. Another 25 exhibited leprosy-like skin lesions at intake, 8 of which were confirmed as having leprosy (50% of which were qPCR positive) and 17 of which were diagnosed with other skin diseases (6% qPCR positive). In skin biopsies, qPCR presented a sensitivity of 0.50 and specificity of 0.94. Furthermore, 955 healthy HHCs were followed-up for at least 3 years and skin scrapings were collected from earlobes for qPCR detection. Positive qPCR indicated a non-significant relative risk of 2.52 of developing the disease. During follow-up, those who progressed towards leprosy exhibited 20% qPCR positivity, compared to 9% of those who remained healthy. Disease-free survival rates indicated that age had a significant impact on disease progression, where patients over 60 had a greater chance of developing leprosy [HR = 32.4 (3.6–290.3)]. Contact tracing combined with qPCR may assist in early diagnosis and age is a risk factor for leprosy progression.

## Introduction

In leprosy, despite the successful introduction of multidrug therapy (MDT), the number of new cases has been slowly decreasing over the past 20 years worldwide. Thus, it has been suggested that MDT is not effective in blocking transmission since a considerable proportion of patients are diagnosed late^1^. It is likely that delay in diagnosis contributes to continuous transmission of mycobacteria, especially to the patients’ household contacts, which is the group that exhibits the highest risk of developing the disease^2–4^.

Active search for new cases among household contacts (HHC) is cost-effective since it can detect patients exhibiting early disease stages^5,6^. Large-scale public policies such chemo and/or immunoprophylactic strategies in HHC are effective^7–9^ in decreasing the disease as a public health problem.

Early leprosy diagnosis is difficult and there is no microbiological, immunological or histopathological test to accurately identify patients. Detection of anti-PGL-I antibodies may assist in the classification of multibacillary patients and monitor treatment efficacy^10,11^. Although positive results indicate higher risk of developing the disease among household contacts, the method presents low sensitivity^10,12^.

Nevertheless, most paucibacillary (PB) patients do not have detectable anti-PGL-I titers^13^. Molecular methodologies are more sensitive and specific than classic techniques such as bacillary load or histopathology^14–16^. In PB patients, PCR exhibited higher specificity and sensitivity when compared to classical methods. Different *M*. *leprae* gene targets derived from different clinical samples such as slit skin smears from earlobes, blood, nasal secretions, and skin/nerve biopsies have been assayed^15,17–20^. The use of qPCR in suspected PB cases improved the sensitivity of leprosy diagnosis at our clinical practice^21,22^.

We, therefore, monitored HHCs at the FIOCRUZ clinic in Rio de Janeiro between 2011–2018 to assess whether the qPCR technique could be used for early diagnosis to detect and confirm the disease among individuals exhibiting suspicious skin lesions and estimate the risk of progression towards disease in a cohort of asymptomatic HHCs. In this study, we confirmed that i) careful clinical examination of HHCs detects leprosy cases at intake; ii) qPCR can improve diagnosis if the contact presents a difficult-to-diagnose skin lesion; iii) the use of qPCR for the screening of asymptomatic contacts is not informative, since only 20% of those who progress are detected, suggesting that qPCR is a not a good predictive marker of disease outcome for this group of incident contacts.

## Results

### A follow-up of household contacts of leprosy patients, 2011–2018

**Figure 1:**
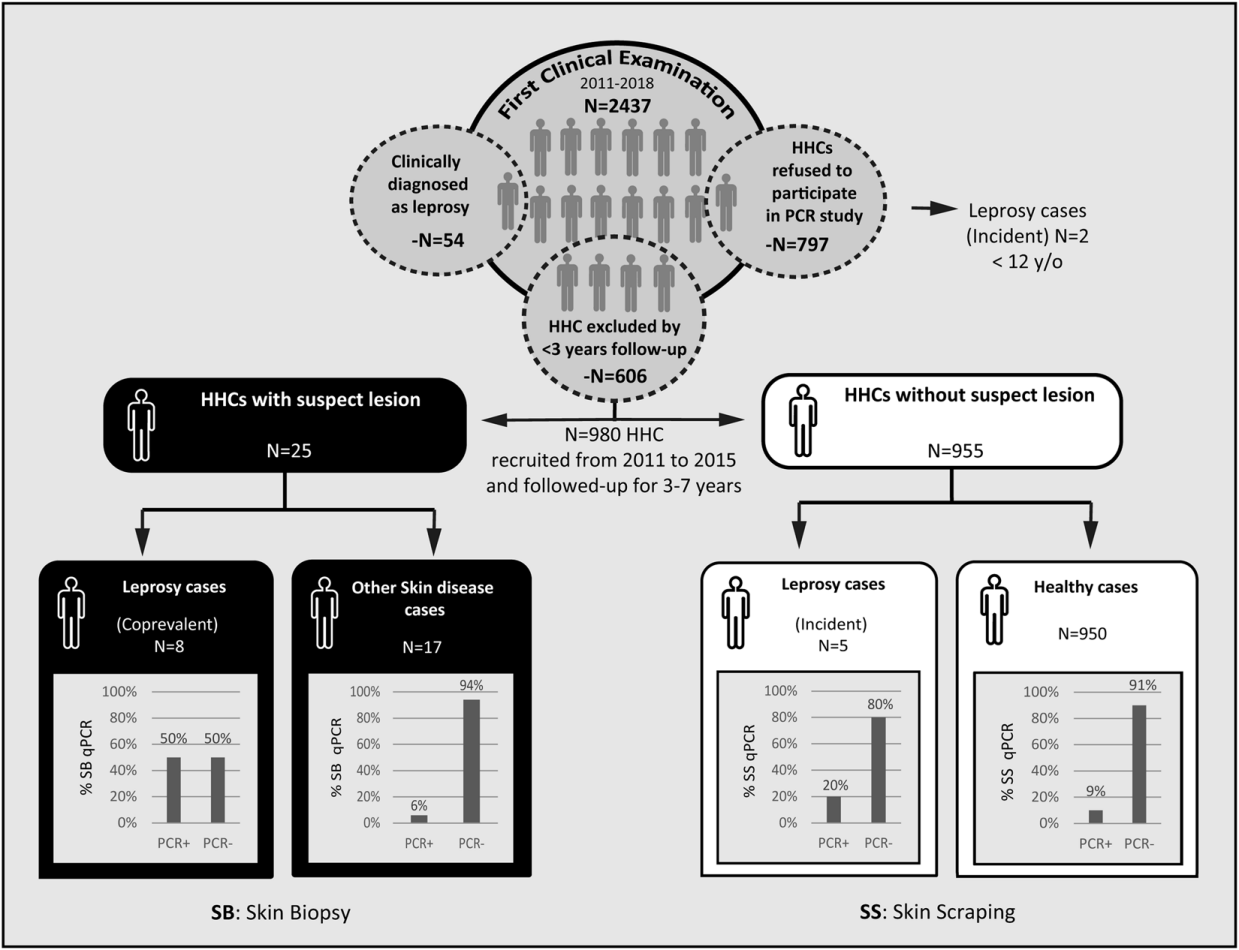
Flowchart of HHC consultations in the Leprosy clinic of the Oswaldo Cruz Foundation between 2011–2018.

Sixty-nine (2.8%) of the 2,437 HHCs examined were either diagnosed as having leprosy during the initial visit or developed leprosy throughout the study (Fig. 1). During the first contact surveillance visit, 54 HHCs (2.2%) were diagnosed with leprosy by clinical evaluation. In a group of 2,383 remaining contacts, 797 refused to donate samples and remain in the study. Of these, 795 remained healthy and 2 (under 12 years of age) developed leprosy during follow-up. Of the 1,586 HCCs, 25 presented difficult-to-diagnose leprosy-like skin lesions and a total of 1,561 HHCs had skin scraping samples from earlobes collected for qPCR between 2011–2018.

### qPCR for early diagnosis of leprosy in suspected patients and for the screening of asymptomatic contacts

The group of 25 suspects were further investigated by 16S qPCR and histopathology in skin biopsies. These laboratory tests confirmed leprosy in 8 patients, while another 17 were diagnosed with other dermatological diseases (ODD). Noteworthy, 50% of these lesions were 16S qPCR positive in leprosy patients (4/8), while only 6% were positive in ODD (1/17) (Fig. 1). Analysis indicates that a positive 16S qPCR result in skin presents an odds ratio of being leprosy (OR = 16, CI = 1.38–185.4). The test had a sensitivity of 50% (CI = 0.14–0.86) and specificity of 94% (CI = 0.69–0.99) when skin biopsies were used for molecular diagnosis of suspect contacts with leprosy-like lesions. For the contact tracing cohort, recruitment was carried out from 2011–2015 and contacts were followed up for a minimum of 3 years and maximum of 7 years. A total of 955 contacts were included and analyzed for 16S qPCR, while five (0.5%) progressed towards leprosy. Only one of these incident cases had a positive 16S qPCR (20%) from the SS sample. Among HHCs who remained healthy after follow-up, 9% were 16S qPCR positive (85/950) (Fig. 1). Relative risk (RR) of predicting leprosy progression in this group was not statistically significant (RR = 2.52; CI = 0.28–22.35).

Accordingly, both qPCR tests showed a high negative predictive value (NPV) of 80% for skin biopsies and 99% for skin scraping. Positive predictive values (PPV) were 80% and 1%, respectively.

### Characteristics of household contacts of leprosy patient cohort

A summary of healthy contacts (n = 950) and contacts who developed leprosy (n = 69) is presented in Table 1. Of these, 54 HHCs were diagnosed with leprosy by clinical evaluation, 8 were further diagnosed with suspicion of leprosy due to leprosy-like lesions and 7 of these contacts developed leprosy during follow-up.

**Table 1:** Clinical and epidemiological characteristics of household contacts of leprosy patient cohort at the Oswaldo Cruz Foundation clinic in Rio de Janeiro, 2011–2018.

Variables	Cohort study healthy contacts with qPCR from SS (n = 950)	Co-prevalent contacts with qPCR from skin biopsy (n = 8)	Co-prevalent contacts without qPCR from skin biopsy (n = 54)	Incident cases with qPCR from SS (n = 5)	Incident cases without qPCR from SS (n = 2)
**Gender**					
Male	382 (40%)	5	20	2	1
Female	568 (60%)	3	34	3	1
**Age**					
1–15 years	95 (10%)	0	12	0	2
16–30 years	259 (27%)	2	10	0	0
31–45 years	319 (34%)	3	12	1	0
46–60	175 (18%)	2	10	0	0
>60 years	102 (11%)	1	10	4	0
**Clinical Form of Index Case**					
TT	21 (2%)	0	0	1	0
BT	240 (25%)	0	10	1	0
BB	115 (12%)	1	1	0	0
BL	269 (29%)	5	7	1	1
LL	243 (26%)	2	35	2	1
I	31 (3%)	0	1	0	0
PN	31 (3%)	0	0	0	0
**Operational Classification of the Index Case**					
MB	627 (66%)	8	43	3	2
PB	323 (34%)	0	11	2	0
**Clinical form of the diagnosed contacts**					
TT	NA	0	5	0	0
BT	NA	6	24	3	1
BB	NA	0	4	0	0
BL	NA	0	4	1	1
LL	NA	0	9	0	0
I	NA	2	7	0	0
PN	NA	0	1	1	0
**Operational Classification of the diagnosed contacts**					
MB	NA	0	16	1	1
PB	NA	8	38	4	1

NA-not applied.

The group presented a 1.42:1 ratio of females to males and an age range of 1 to 90 years.

Among the 69 contacts who were diagnosed with leprosy, eighteen co-prevalent contacts (0.7%) were diagnosed with MB leprosy and 51 (2.1%) presented PB leprosy. Two incident contacts (0.1%) progressed towards MB leprosy and 5 (0.2%) developed PB leprosy. Eighty percent (56/69) of the contacts diagnosed with leprosy had a multibacillary case as the index patient. Among contacts who were diagnosed with leprosy at the initial visit or developed leprosy during the study (69/2,437), 74% (51/69) were diagnosed with PB forms of leprosy.

**Figure 2:**
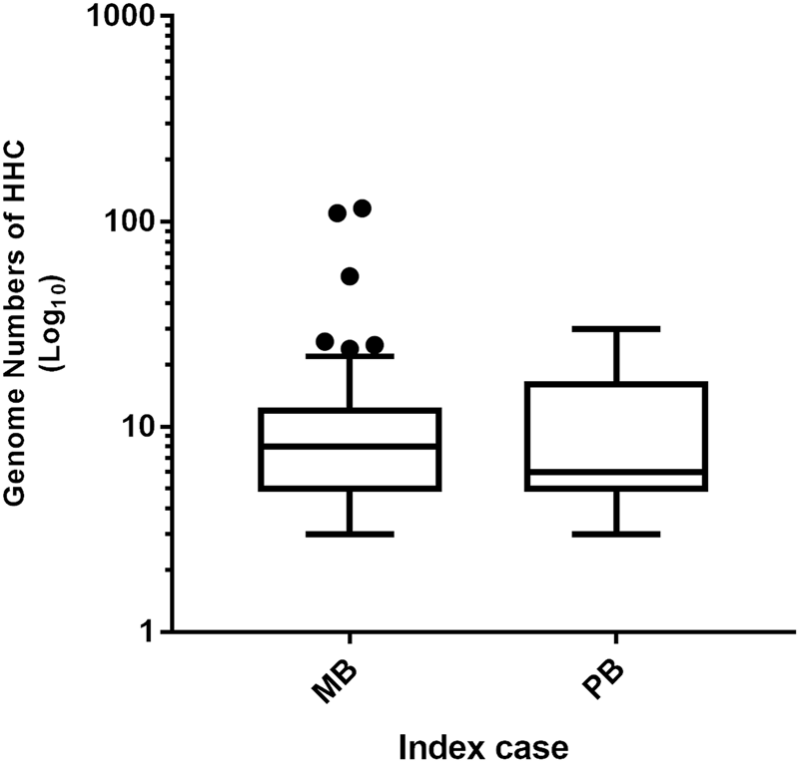
Comparison of SS qPCR positive samples: *M*. *leprae* genome counts in household contacts stratified according to index patients. Box-plot presenting the number of genomes from PCR positive HCC skin scraping in accordance with the operational classification of the index cases. PB = Paucibacillary (n = 26); MB = Multibacillary (n = 60). Each point represents the individual outlier value of a genome count. Within each box, the median, represented by the middle horizontal lines, was 6 for PB and 8 for MB cases.

Of the 955 subjects included in the cohort study, 9% were positive for SS 16S qPCR. These were stratified according to the clinical form of the index case, a positivity rate of 8.1% for HHCs of PB cases and 9.4% for MB index cases. Additionally, considering only HHC samples that were 16S qPCR positive in the cohort study, the median genome count did not vary between PB and MB index patients (6 and 8, respectively - Fig. 2), even though a few contacts were shown to have high genome counts (between 25 and 100 genomes). Among the five contacts who progressed towards disease, the only contact who presented a positive SS 16S qPCR had a genome count of 7 genomes. Despite careful follow-up of the HHCs with higher genome counts, none of them developed leprosy over the following 3 years. Among the five incident cases, 80% were diagnosed with PB forms of leprosy.

### The impact of qPCR and other clinical/demographic features on HHC prognosis

**Figure 3:**
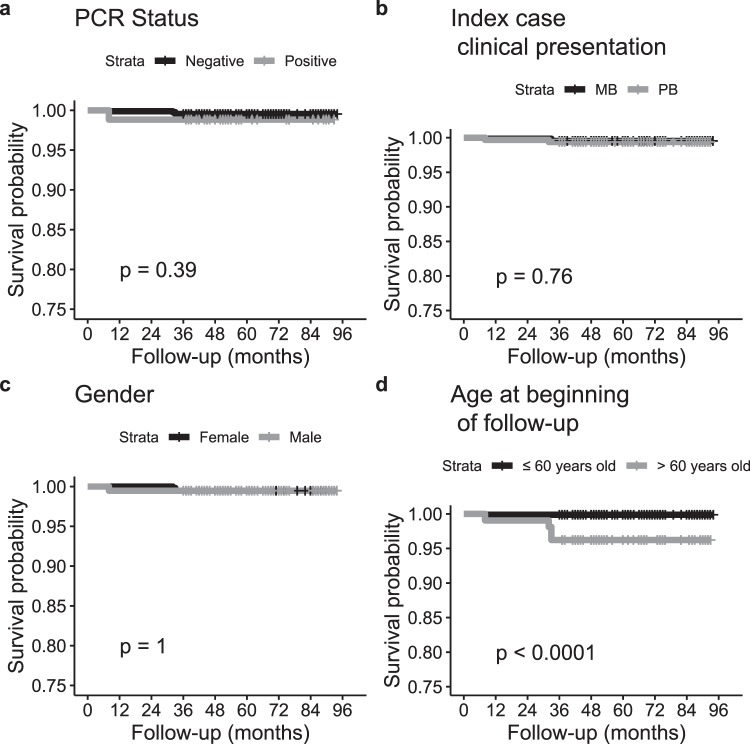
Kaplan-Meier plots of disease-free survival variables: PCR results (**a**), index case classification (**b**), gender (**c**) and age at the beginning of the follow up (**d**) of HHCs of the cohort study. To assess differences between curves, the Log Rank test was performed and p < 0.05 was considered significant.

Disease-free survival rates were analyzed for the respective variables: qPCR, age at the beginning of follow-up, index case classification and gender (Fig. 3). For this, we analyzed hazard ratio (HR), which is a measure of an effect of an intervention on an outcome over time. Data indicates that age at the beginning of follow-up had a significant impact on survival. Subjects over 60 years of age had a higher chance of progressing toward leprosy than younger subjects with a hazard ratio (HR) of 32 (CI = 3–290). This is an important result that could help manage contact surveillance despite low precision estimation due to the small number of incident cases.

## Discussion

In this work, qPCR was used in two different strategies: i) to verify whether 16S qPCR could be employed to support diagnostic confirmation in household contacts with suspicious skin lesions; and ii) to test among healthy individuals whether positivity could be used as predictive markers of disease progression. Unfortunately, the use of SS 16S qPCR in earlobes of healthy contacts does not appear to be a good predictive marker of disease progression. Nevertheless, a positive 16S qPCR in a skin biopsy can be considered an important factor in the diagnosis of leprosy when household contacts present leprosy-like skin lesions. In this case, analysis confirmed that a positive qPCR test determines a high probability of having leprosy with high specificity and reasonable sensitivity. In fact, 50% of 16S qPCR positive samples were confirmed as leprosy cases. Therefore, data indicates that qPCR can be used for routine screening among difficult-to-diagnose suspected patients. We also observed that most contacts (74%) who had leprosy at the initial visit or progressed to leprosy during the study were diagnosed with PB forms of leprosy.

In this work, we confirm our previous findings in which the use of qPCR was shown to improve precision of diagnosis in PB leprosy^21–23^.

On the other hand, when we followed up a population of healthy contacts, we observed that 9% tested positive for *M*. *leprae* DNA. Indeed, few studies reported PCR positivity using SS from earlobes, however, when screening contacts using nasal swabs, positivity ranges from 4 to 16%^24–26^, while peripheral blood cell qPCR detection is lower (1.2%)^20,27^. Different biological samples and *M*. *leprae* targets can affect the variability of PCR positivity in contacts of leprosy patients. In this sense, the use of multi-copy targets such as RLEP, which is more sensitive, could affect test performance. However, in this work, we chose to use the 16S single copy target because it presents higher specificity based on the group’s previous results^23^. Indeed, the sensitivity of the 16S qPCR test in the SS of household contacts was low (20%), but higher than previously published in other studies^20,24–26^.

More recently, PCR screening in the blood of PGL-I positive contacts increased the rate of PCR positivity, having found 40% of qPCR positivity in this group. Nevertheless, authors fail to provide a follow-up to evaluate those who progressed towards leprosy^28^. Our data clearly demonstrate that qPCR alone is not a good estimate to infer the risk for a contact to develop leprosy. Nevertheless, we observed a higher positivity of the 16S qPCR from SS from contacts of MB patients and did not detect significant differences in the number of genomes in contacts of PB or MB index patients. Thus, our data indicate that there is no correlation between the level of exposure as measured by index cases and 16S qPCR positivity that could be assumed as infection. Therefore, genome number failed to predict disease progression, even among individuals bearing a high number of genomes. Improving qPCR sensitivity, better DNA extraction methods and testing of new qPCR markers may provide better estimates, although the cost-effectiveness of these laboratory tests also needs to be assessed.

Literature suggests that the risk of developing the disease is higher among HHC that present a multibacillary index patient and that a higher prevalence was detected among those aged 15 and older^2–4,27,29,30^. These results are probably due to the long incubation period of the leprosy bacillus subsequent to or combined with lengthy periods of exposure. Although this study is a follow-up of contacts of leprosy patients for at least 3 and a maximum of 7 years, the total number of individuals who developed the disease was low, which indicates the need for a longer follow-up period to generate more robust results. Nevertheless, our data shows that contacts over 60 years of age have a high risk of progressing to disease, which could suggest the need for a specific surveillance and monitoring policy directed towards this age group.

Chemo- and immunoprophylactic protocols have been tested over the past few years. Our work shows that careful dermato-neurological examination along with the use of qPCR screening for those with leprosy-like lesions could aide in precise and early diagnosis. Thus, all other HHCs without skin lesions could be included in chemo- and/or immunoprophylactic regimens. We still need a test to narrow down high-risk contacts who would need chemoprophylactic treatment in a way that we could preclude unnecessary treatments to healthy individuals who are not under risk of developing leprosy^8^. Chemoprophylactic protocols should be followed very carefully by contacts from leprosy patients since the duration of this protection and the cost-effectiveness is always an issue concerning the targeted audience^31^. Indeed, new predictions suggest that a test which improves new case detection rate by 50% could impact leprosy transmission^12,32^. Herein, 16S qPCR showed 20% positivity among contacts who progress to disease, while PGL-I revealed poorer results^10,31^. So far, 4 out of 5 cases were negative for qPCR, which is a low number of individuals who progressed to the disease to draw a definite conclusion. Nevertheless, it could indicate that preventive measures based on qPCR results would miss most of the individuals at risk within this group. In the absence of novel markers, the use of prophylactic protocols is indicated for all contacts, as has been suggested previously.

In summary, we recently found that the availability of qPCR in the routine of our clinic improved the ability to diagnose leprosy early in patients suspected of PB leprosy^22^. Our study corroborates the fact that the epidemiological surveillance of HHC is extremely important for early detection of the disease, although the use of qPCR does not help to predict leprosy progression. The standardization and implementation of more sensitive, accurate and effective tools to help confirm diagnosis is necessary in this group of household contacts.

## Methods

### Ethics statement

This study was approved by the FIOCRUZ ethics committee (CEP: 976.330) and was in accordance with the Helsinki Declaration. An informed consent form was signed by patients, HHCs, and parents or guardians of children under 18 years of age who were included in the study.

### Study design

The study was conducted between 2011 and 2018 at the Leprosy clinic of the Oswaldo Cruz Foundation in the city of Rio de Janeiro, Brazil as summarized in Fig. 1. All registered HHCs from each newly diagnosed patients were invited to participate in the study. An informed consent form was signed and the HHCs underwent dermatological and neurological examinations. When skin lesions were identified during this first visit, HHCs were either i) clinically diagnosed as having leprosy or ii) investigated as a leprosy suspect case from which a biopsy was taken for histopathology and qPCR. Contacts diagnosed at intake, either by clinical or laboratory examination, were called “co-prevalent cases”. All asymptomatic contacts examined at intake were invited to be part of the follow-up (cohort) study for at least 3 years and at most 7 years.

If they agreed to participate, a skin scraping from the right earlobe was collected for qPCR detection. HHCs who developed the disease during follow-up (2011–2018) were considered incident cases.

For data analysis, HHCs were stratified according to operational [paucibacillary (PB) or multibacillary (MB)] and clinical classifications of their index cases. PB individuals were classified as Tuberculoid (TT), Borderline tuberculoid (BT), Indeterminate (I) and Pure Neural (PN). MB individuals were classified as Borderline–borderline (BB), Borderline lepromatous (BL) or Lepromatous (LL)^33^. Contacts with clinically diagnosed leprosy were also stratified according to clinical and operational classifications. Sociodemographic variables including age and gender were collected.

### Sample collection

Skin scraping (SS) from all recruited household contacts were collected at the first appointment. SS was performed by a small incision on the right earlobe assisted by a razor blade followed by scraping of the region under tweezer pressure. Samples were stored in 70% ethanol at −20 °C until processing. If a household contact presented patches, nodules or macula during dermatological examination, a skin biopsy was collected using a 6-mm punch. Biopsies were split into halves and fixed in 10% formalin for routine H&E staining and/or 70% ethanol for DNA extraction and qPCR as described below. Ethanol-stored skin samples were frozen and placed in liquid nitrogen.

### Histopathology

Biopsy samples obtained from HHCs with skin lesions suspected of being leprosy were fixed in 4% formaldehyde, dehydrated, clarified with xylene and embedded in paraffin for routine histopathological examination. The paraffin blocks were sectioned into 5-µm-thick sections with a microtome and stained with haematoxylin-eosin and Wade’s stain for acid-fast bacilli. Histopathological slides were analyzed by pathologists and diagnosis was confirmed when histological features of leprosy were observed and cases were classified according to Ridley & Jopling^33^.

### DNA extraction

DNA from ethanol-stored skin biopsies or SS samples was extracted using DNeasy Blood and Tissue Kit - QIAGEN®. Briefly, samples were centrifuged at 2,000 rpm for 10 minutes. Extraction was performed according to manufacturer’s protocols (QIAGEN). Eluted DNA concentration was determined with a NanoDrop 1000 Spectrophotometer (Thermo Scientific).

### Detection of M. leprae DNA by 16S qPCR

For SS or skin biopsy samples, qPCR was used to amplify *M*. *leprae* specific 16S rRNA target using the TaqMan amplification assay^34^. All reactions were performed in triplicate on the same real-time PCR system (StepOne, Applied Biosystems). Titrated *M*. *leprae* DNA was used as a positive control while water was used in negative controls (MIQE is provided as Supplement Table). A cycle threshold value of 0.05 was used to define positive samples as described previously, and the sample was considered positive when it exhibited Cq</=38.5 (3 genomes) in at least two out of three triplicate reactions^22^. The number of genomes was calculated by interpolating sample Cq values with those from a dilution curve made from a known number of *M*. *leprae* genomes^21,23^.

### Statistical analysis

Data were used to calculate Odds Ratio (OR) and relative risk (RR) for clinically confirmed leprosy using OpenEpi. Sensitivity, specificity, positive predictive value, and negative predictive value of qPCR were also calculated. Confidence intervals were calculated at a 95%. To assess the impact of PCR and other clinical/demographic features on HHC prognosis, disease-free survival analyses were performed. Kaplan-Meier curves were designed, and log-rank test was applied to compare curves. All these analyses were carried out using the survival package within the R environment.

## Supplementary information

Supplementary table 1
