# Supplementary material for: Quantitative PCR for leprosy diagnosis and monitoring in household contacts: A follow-up study, 2011–2018
Source: Sci Rep. 2019 Nov 13;9:16675. doi: 10.1038/s41598-019-52640-5 (PMC6854052; doi:10.1038/s41598-019-52640-5)
Supplement: Supplementary file 1 — Supplementary table 1 [file 41598_2019_52640_MOESM1_ESM.pdf]

Quantitative PCR for leprosy diagnosis and monitoring in household contacts: A follow-up study, 2011-2018.

Fernanda S.N. Manta, Raquel R. Barbieri, Suelen J. M. Moreira, Paulo T. S. Santos, José A. C. Nery, Nádia C. Duppre, Anna M. Sales, Antônio G. Pacheco, Mariana A. Hacker, Alice M. Machado, Euzenir N. Sarno, Milton O. Moraes.

S1Table - MIQE Checklist.

Item to check	Importance	Experimental design
Definition of experimental and control groups	E	**Experimental group**: Household Contacts from each newly diagnosed patients registered between 2011 and 2018 at the Leprosy clinic of the Oswaldo Cruz Foundation in the city of Rio de Janeiro, Brazil.
Number within each group	E	**Experimental group:** 980 HCCs
Assay carried out by the core or investigator's laboratory?	D	Yes
Acknowledgment of authors' contributions	D	
**Sample**		
Description	E	**Experimental samples**: Skin scraping was performed by a small incision on the right earlobe for each HHC and a skin biopsy was collected If a household contact presented patches, nodules or macula during dermatological examination.
Volume/mass of sample processed	D	Skin scraping – dermal cells or skin biopsy from 6-mm punch
Microdissection or macrodissection	E	
Processing procedure	E	Skin Scraping was performed by a small incision on the right earlobe assisted by a razor blade followed by scraping the region under tweezer pressure, collected and stored in 70% ethanol. If a household contact presented patches, nodules or macula during dermatological examination, a skin biopsy was collected using a 6-mm punch.
If frozen, how and how quickly?	E	Samples were frozen in -20 ºC immediately after they were obtained.
If fixed, with what and how quickly?	E	Not fixed
Sample storage conditions and duration	E	Samples were stored in 70% ethanol at -20ºC until processing.
**Nucleic acid extraction**		
Procedure and/or instrumentation	E	DNA from ethanol-stored skin biopsies or SS samples was extracted using DNeasy Blood and Tissue - QIAGEN®. Briefly, samples were centrifuged at 2,000 rpm for 10 minutes. Extraction was performed according to manufacturer's protocols (QIAGEN). The purified DNA was dissolved in 100ul and stored at -20°C.
Name of kit and details of any modifications	E	DNeasy Blood and Tissue - QIAGEN®. We followed manufacture's protocol.
Source of additional reagents used	D	Ethanol (Merck)
Details of DNase or RNase treatment	E	
Contamination assessment (DNA or RNA)	E	Negative controls (tube without DNA) were performed in order to assess the absence of DNA contamination in the process.
Nucleic acid quantification	E	DNA concentration was determined by measuring the absorbance at 260 nm UV light
Instrument and method	E	Eluted DNA concentration was determined with a NanoDrop 1000 Spectrophotometer (Thermo Scientific).
Purity (A260/A280)	D	DNA purity was determined by measuring the absorbance ratio 260/280
Yeld	D	Skin scraping provide 5-20ng/ul of total DNA; skin biopsies show 100-500ng/ul
RNA integrity: method/instrument	E	
RIN/RQI or Cq of 3' and 5' transcripts	E	
Electrophoresis traces	D	
Inhibition testing (Cq dilutions, spike, or other)	E	

Reverse transcription		
Complete reaction conditions		

	E	
Amount of RNA and reaction volume	E	
Priming oligonucleotide (if using GSP) and concentration	E	
Reverse transcriptase and concentration	E	
Temperature and time	E	
Manufacturer of reagents and catalogue numbers	D	
Cqs with and without reverse transcription	D	
Storage conditions of cDNA	D	
**qPCR**		
Complete reaction conditions	E	The levels of M. leprae 16S rRNA in skin biopsy specimens were estimated using TaqMan qPCR amplification. Purified total DNA (10 ng) in 5 ml were added to a total PCR reaction volume of 25 ul containing TaqMan master mix (Applied BioSystems), 500 nM of each primer and 100 nM of probe for16S rRNA PCR assays.<br>Reaction mixtures were prepared in triplicates and subjected to 50°C for 2 min, 95°C for 10 min, and 40 cycles of 95°C for 15 sec and 60°C for 1 min using a StepOne real-time PCR system (Applied BioSystems).<br>A cycle threshold value of 0.05 was used to define positive samples, and the sample was considered positive when it exhibited Cq </= 38.5 in at least two out of three triplicate reactions.
Reaction volume and amount of cDNA/DNA	E	Reaction volume: 25 ul; 50ng DNA
Primer, (probe), Mg2, and dNTP concentrations	E	500 nM Primers; and 100 nM Probe
Polymerase identity and concentration	E	TaqMan Universal Master Mix II with UNG (Thermo Fisher Scientific), 2X
Buffer/kit identity and manufacturer	E	TaqMan Universal Master Mix II with UNG (Thermo Fisher Scientific), 2X
Exact chemical composition of the buffer	D	
Additives (SYBR Green I, DMSO, and so forth)	E	
Manufacturer of plates/tubes and catalog number	D	MicroAmp Fast 96-Well Reaction Plate (0.1 mL), cat:4346907
Complete thermocycling parameters	E	50°C for 2 min, 95°C for 10 minute, then 40 cycles at 95°C for 15 seconds, 60°C for 1 minute.
Reaction setup (manual/robotic)	D	Manual
Manufacturer of qPCR instrument	D	StepOne real-time PCR system (Applied BioSystems).

**qPCR validation**		
Evidence of optimization (from gradients)	D	
Specificity (gel, sequence, melt, or digest)	E	
For SYBR Green I, Cq of the NTC	E	
Calibration curves with slope and y intercept	E	
PCR efficiency calculated from slope	E	
CIs for PCR efficiency or SE	D	
r2 of calibration curve	E	
Linear dynamic range	E	
Cq variation at LOD	E	
CIs throughout range	D	
Evidence for LOD	E	
If multiplex, efficiency and LOD of each assay	E	

**Data analysis**		
qPCR analysis program (source, version)	E	Step One Software v.2.3
Method of Cq determination	E	The threshold is determined using the Amplification-based Threshold method. The threshold is used to specify Cq values of samples.

Outlier identification and disposition	E	A cycle threshold value of 0.05 was used to define positive samples.
Results for NTCs	E	The signal of the amplification plot was undetermined (NA).
Justification of number and choice of reference genes	E	The target selection was based on the specificity and sensitivity levels previously found by the group and already published (Martinez., 2011).
Description of normalization method	E	
Number and concordance of biological replicates	D	>66%
Number and stage (RT or qPCR) of technical replicates	E	All samples were analyzed in triplicate
Repeatability (intraassay variation)	E	
Reproducibility (interassay variation, CV)	D	
Power analysis	D	
Statistical methods for results significance	E	
Software (source, version)	E	
Cq or raw data submission with RDML	D	
**qPCR target information**		
Gene symbol	E	16S
Sequence accession number	E	NC_002677.1
Location of amplicon	D	1341345-1341415
Amplicon length	E	71bp
In silico specificity screen (BLAST, and so on)	E	BLAST
Pseudogenes, retropseudogenes, or other homologs?	D	
Sequence alignment	D	
Secondary structure analysis of amplicon	D	
Location of each primer by exon or intron (if applicable)	E	
What splice variants are targeted?	E	
**qPCR oligonucleotides**		
Primer sequences	E	ML16S rRNATaq-F: 5-GCA TGT CTT GTG GTG GAA AGC-3<br>ML16S rRNATaq-R: 5-CAC CCC ACC AAC AAG CTG AT-3
RTPrimerDB identification number	D	
Probe sequences	D	ML16S rRNATaq-Probe 5_CAT CCT GCA CCG CA-3_
Location and identity of any modifications	E	No modifications were done
Manufacturer of oligonucleotides	D	Thermo Fisher Scientific
Purification method	D	Desalted

Legend: (E) essential and (D) desirable information to be included in research reports using qPCR.
